# Two Naturally Occurring Terpenes, Dehydrocostuslactone and Costunolide, Decrease Intracellular GSH Content and Inhibit STAT3 Activation

**DOI:** 10.1371/journal.pone.0020174

**Published:** 2011-05-18

**Authors:** Elena Butturini, Elisabetta Cavalieri, Alessandra Carcereri de Prati, Elena Darra, Antonella Rigo, Kazuo Shoji, Norie Murayama, Hiroshi Yamazaki, Yasuo Watanabe, Hisanori Suzuki, Sofia Mariotto

**Affiliations:** 1 Department of Life and Reproduction Sciences, University of Verona, Verona, Italy; 2 Department of Pharmacology, Showa Pharmaceutical University, Machida, Tokyo, Japan; 3 Department of Medicine, University of Verona, Verona, Italy; 4 Department of Pharmacokinetics, Showa Pharmaceutical University, Machida, Tokyo, Japan; The University of Kansas Medical Center, United States of America

## Abstract

The main purpose of the present study is to envisage the molecular mechanism of inhibitory action ofdehydrocostuslactone (DCE) andcostunolide (CS), two naturally occurring sesquiterpene lactones, towards the activation of signal transducer and activator of transcription 3 (STAT3). We report that, in human THP-1 cell line, they inhibit IL-6-elicited tyrosine phosphorylation of STAT3 and its DNA binding activity with EC_50_ of 10 µM with concomitantdown-regulation ofthe phosphorylation of the tyrosine Janus kinases JAK1, JAK2 and Tyk2. Furthermore, these compounds that contain an α-β-unsatured carbonyl moiety and function as potent Michael reaction acceptor, induce a rapid drop in intracellular glutathione (GSH) concentration by direct interaction with it, thereby triggering *S*-glutathionylation of STAT3. Dehydrocostunolide (HCS), the reduced form of CS lacking only the α-β-unsaturated carbonyl group, fails to exert any inhibitory action. Finally, the glutathione ethylene ester (GEE), the cell permeable GSH form, reverts the inhibitory action of DCE and CS on STAT3 tyrosine phosphorylation. We conclude that these two sesquiterpene lactones are able to induce redox-dependent post-translational modification of cysteine residues of STAT3 protein in order to regulate its function.

## Introduction

The Janus kinases (JAKs) and signal transducers and activators of transcription (STATs) pathway is responsible for signal transduction of a large number of cytokines and growth factors that regulate responses to inflammation or immune challenge and address the future of cell decision during development and neoplastic growth [1]. After the cytokines binding to their cognate receptors, JAK tyrosine kinases, JAK1, JAK2 and Tyk2, are promptly activated and proceed to tyrosine-phosphorylate cytosolic, latent STATs. The phosphorylated STATs homo- or hetero-dimerize and translocate into the nucleus to regulate expression of target genes. In addition to tyrosine phosphorylation, STATs are occasionally phosphorylated on serine residues, located on their carboxyl-terminal trans-activation domains [2].

Seven STATs proteins have been identified so far. In physiological conditions STATs activation is transient and usually lasts from a few minutes to several hours. This is assured by a variety of negatively acting events thatblock further activation, decrease DNA binding or result indephosphorylation of STATs. The final outcome of cytokine/growth factor stimulation reflects how different cell types can interpret the complex and often contrasting signals they receive.

The pleiotropic cytokine IL-6 activates predominantly STAT3 binding to Gp130 receptor complex and modulate the expression of genes encoding mediators crucial for the classic physiological acute phase response and for the apoptotic pathway [3]. Although tyrosine phosphorylation of STAT3 is accompanied by serine phosphorylation in a variety of cells, the biologic role of serine STAT3 phosphorylation is controversial. Some studies suggest that serine phosphorylation enhances transcriptional activity [4], whereas other reports demonstrate that serine phosphorylation induces inhibitory activity [5], [6].

Although STAT3 activation normally leads to the physiological response, deregulation of this transduction cascade could lead to the tissue damage and directly or indirectly could be involved in different pathologies. A number of inflammation-correlated diseases such as Crohn's disease, pleurisy, psoriasis etc. are characterized by hyperactivationof STAT3 [7], [8], [9]. Furthermore STAT3 is considered as an oncoprotein and its constitutive activation is reported in numerous solid and haematological tumours [10]. Therefore, any treatment counteracting the hyper-expression or -activation of STAT3 has been considered as a new strategy to treat these world-widely increasing pathologies [11], [12]. In this context, the use of naturally occurring compounds, especially those present in plants, has recently attracted the attention of many researchers. We recently reported that green tea extract or epigallocatechin-3-gallate, the main green tea component, and hyperforin present in St. John Wort extract exert a strong inhibitory action on IFN-γ-elicited STAT1 activation, indicating the possibility of their use in the prevention/therapy against stroke and diabetes, respectively [13], [14], [15].

The cellular redox state is a crucial mediator of multiple metabolic, signalling and transcriptional processes in cells, and a fine balance between oxidizing and reducing conditions is essential for normal function and survival of cells [16]. The disturbance in the glutathione/glutathione disulphide couple (GSH/GSSG) homeostasis is implicated in the ethiology and/or progression of a number of human diseases, including cancer, neurodegenerative diseases, cystic fibrosis etc. [17]. GSH deficiency manifests itself largely through an increased susceptibility to oxidative stress, and the resulting damage is a key step in the onset and progression of many disease states. Conversely, elevated GSH levels generally increase antioxidant capacity and resistance to oxidative stress, and this is observed in many types of cancer cells. Oxidative stress may cause reversible and/or irreversible oxidative modifications on sensitive proteins that may lead to a change in their activity or function [18]. Mild oxidative stress induces reversible modifications, including the glutathionylation of cysteine residues. This reaction may have a dual role: protection from irreversible damage and modulation of protein function. Conversely, excessive oxidative stress triggers irreversible modification of thiolic groups of proteins generally associated with permanent loss of function, misfolding and aggregation [19]. Recent report indicates that STAT3 can be glutathionylated under oxidative conditions with concomitant inhibition of its phosphorylation, thus suggesting the possible cross-talk between these two post-translational reactions [20].

Dehydrocostuslactone (DCE) and costunolide (CS), two sesquiterpene lactones present in a number of plants such as *Laurusnobilis L., Magnolia sieboldii L. and Sassureacustus L.*, exhibit various biological and immunological activity, including antinflammatory and antifungal one [21], [22]. It has also been reported the proapoptotic effect of these compounds in different human cancer cells [23], [24], [25], [26]. These compounds exert an inhibitory action on NF-κB pathway [27], [28], induce Nfr2 activation [29], [30] and activate many MAP kinases such as JNK, ERK2 and P38 [23]. Moreover, DCE inhibits the constitutive STAT3 activation through an increase in suppressor of cytokine signalling (SOCS)-1 and (SOCS)-3 expression [31]. However, the main target of the molecular mechanism of their biological action has not been clarified so far.

In the present study we hypothesized that decrease in the intracellular GSH level, induced by DCE and CS, triggers the inhibition of STAT3 tyrosine phosphorylation mediated by its glutathionylation. To evaluate this, we examined the effect of two terpenes on the activation of STAT3 induced by IL-6 in human acute monocyticleukemia THP-1 cells. We present data indicating the direct involvement of GSH deficiency in the inhibition of STAT3 pathway through the glutathionylation of STAT3.

## Materials and Methods

### Chemicals

**Figure 1:**
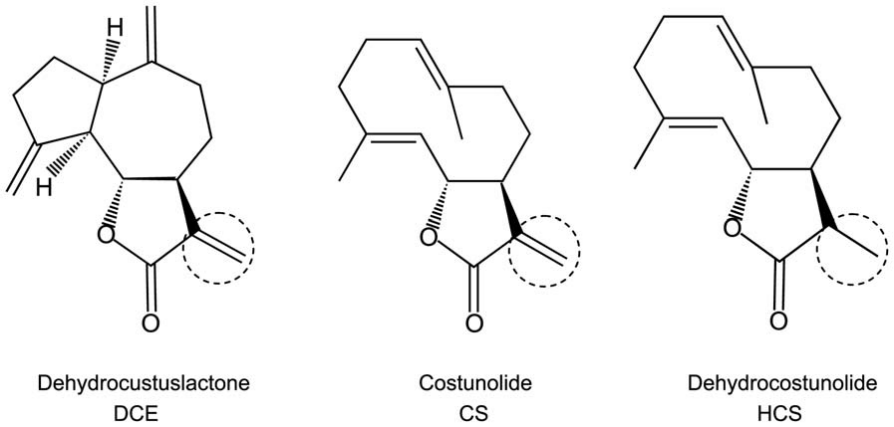
Structure of Dehydrocostuslactone (DCE), Costunolide (CS) and Dehydrocostunolide (HCS). The α-β-unsaturated carbonyl group is marked with a dotted circle.

All chemicals used throughout the present study were of the highest analytical grade, purchased from Sigma Chemical Company, Milan, Italy, unless otherwise specified. RPMI 1640 medium, Dulbecco's modified Eagle's medium (DMEM) and fetal bovine (FBS) serum were obtained from Lonza, Verviers, BE. DCE and CS (Figure 1) were purchased from PhytoLab GmbH & Co, Vestenbergsgreuth, Germany.

Rabbit anti-phospho-Tyr^705^STAT3, anti-phospho-Ser^727^STAT3, anti-phospho-Tyr^1054/55^TyK2 and anti-JAK2 were purchased from Cell Signaling Technology, Beverly, MA; rabbit anti-phospho-Tyr^1022/1023^JAK1 and anti-phospho-Tyr^1007/1008^JAK2 antibodies were obtained from Millipore, Bedford, MA; rabbit anti-SOCS-3 was from ImmunoBiological Laboratories, Tokyo, Japan; rabbit anti-STAT3, anti-Tyk2 and anti-JAK1 antibodies werefrom Santa Cruz Biotechnology, Inc., Santa Cruz, CA.

### Cell culture

Human monocyticleukemia THP-1 cells (American Type Culture Collection, Manassas, VA) were cultured in RPMI 1640 supplemented with 10% FBS, 100 UI/ml penicillin, 100 µg/ml streptomycin and 40 µg/ml gentamycin in a 5% CO_2_ atmosphere at 37°C. Human cervical carcinoma HeLa cells (American Type Culture Collection) and human colorectal adenocarcinoma DLD-1 cells (American Type Culture Collection) were cultured in DMEM supplemented with 10% FBS under the same conditions.

### Construction of Tyk2

Tyk2 was amplified by PCR with the following primer sets:

5′-CG**GAATTC**ATGCCTCTGCGCCACTGG-3′ (sense)
5′-AC**GCGGCCGC**TCAGCACACGCTGAACAC-3′ (antisense).

PCR primers included 5′-NotI, 5′-EcoRI restriction sites (boldface) to aid cloning. PCR products were digested completely with NotI, EcoRI, gel-purified, and insert into the identical sites of pcDNA 3.0 to give Tyk2-pcDNA 3.0. The nucleotide sequence of this expression plasmid was verified by DNA sequencing.

### Cells transfection

For a transient transfection, HeLa cells were plated into 60-mm plates at a density of 8×10^5^ in DMEM without antibiotics. After 18 hours, the DMEM was replaced with the serum-reduced medium OPTI-MEM (Invitrogen, Carlsbad, CA) and the cells were transfected with 1 µg Tyk2-pcDNA 3.0 expression vector and 10 µllipofectamine 2000 according to the manufacturer's instructions (Invitrogen, Carlsbad, CA). After 24 hours, the cells were treated with IL-6 (20 ng/ml) for 5 minutes in order to achieve Tyk2 activation. Effect of two terpenes on TyK2 activation was examined by treating cells for 30 minutes before IL-6 stimulation. The cells were harvested and used for Western blot analysis.

### Electrophoretic Mobility Shift Assay-EMSA

3×10^6^ THP-1 or DLD-1 cells, after 4 hours starvation, were treated with IL-6 (20 ng/ml) for 15 minutes in order to achieve STAT3 activation. Effect of two terpenes on STAT3 activation was examined by treating cells for 30 minutes before IL-6 stimulation. Nuclear extracts of THP-1 cells were prepared according to Osborn et al. [32] in the presence of 10 µg/ml leupeptin, 5 µg/ml antipain, 5 µg/ml pepstatin, and 1 mM phenylmethylsulfonyl fluoride. Eight micrograms of nuclear extract were incubated with 2–5×10^4^ cpm of ^32^P-labeled double-stranded oligonucleotides, the consensus STAT3 DNA binding site (sis-inducible factor-binding recognition element, SIE/m67) from the c-fos promoter (5′-gtcgaCATTTCCCGTAAATCg-3′), in a 15 µl reaction mixture containing 20 mM Hepes, pH 7.9, 50 mM KCl, 0.5 m Mdithiothreitol, 0.1 mM EDTA, 2 µg of poly(dI-dC), 1 µg of salmon sperm DNA, and 10% glycerol. Products were fractionated on a non-denaturing 5% polyacrylamidegel. The gels were dried and autoradiographed and the intensity of hybridization was quantified using the public domain NIH Image 1.61 program (developed at the U.S. National Institutes of Health and available on http://rsb.info.nih.gov/nih-image/). Supershift assay was performed by incubating the nuclear extracts in a binding buffer for 1 hour at 4°C with 1 µl of antibody before addition of labelled oligonucleotide.

### Western blot analysis

Cells, treated as described above, were homogenized at 4°C in 20 mM HEPES, pH 7.4, containing 420 mM NaCl, 1 mM EDTA, 1 mM EGTA, 1% Nonidet-P40 (NP-40), 20% glycerol, protease cocktail inhibitors (GE Healthcare, Amersham Place, UK) and phosphatase cocktail inhibitors. Aliquots of the cell lysate (40 µg total protein/lane) were loaded on 7.5% SDS-polyacrylamide gels. Electrophoresis was performed at 100 V with a running buffer containing 0.25 M TrisHCl, pH 8.3, 1.92 M glycine, and 1% SDS. The resolved protein were electroblotted onto a PVDF membrane (Immobilon P, Millipore, Bedford MA) and incubated overnight at 4°C with the indicated primary antibodies. After washing, membraneswere developed using anti-rabbit or anti-mouse IgG peroxidase-conjugated antibody (Cell Signaling Technology) and chemiluminescent detection system (Immun-Star™ WesternC™ Kit, Bio-Rad, Hercules, CA). Blotted proteins were detected and quantified using the ChemiDoc XRS Imaging System (Bio-Rad, Hercules, CA). After stripping, membranes were re-hybridized with the correspondent antibodies.

### GSH and GSSG recycling assay

The intracellular oxidized and reduced glutathione were determinedspectrophotometrically using the GSH recycling method as previously described [33]. Briefly, treated and untreated cells werelysed by freezing and thawing in 100 mM sodium phosphate buffer, pH 7.5, containing 5 mM EDTA, (KPE buffer) and after centrifugation at 16,000 rpm for 10 minutes, total protein concentration was determined by using Bradford method [34]. The supernatant was removed and deproteinized with 5% trichloroacetic acid (TCA). For total GSH measurement (GSH+GSSG), sample aliquot of TCA extract was incubated in 1 ml of KPE buffer containing 0.6 mM 5,5′-dithio-bis-(2-nitrobenzoic acid) (DTNB) and 1 U/ml GSH reductase. The reaction was startedby the addition of 0.2 mM NADPH and the increase in absorbance at 412 nm was measured. The amount of total GSH was determined by comparison with GSH standard curve. For the determination of GSSG, the same DTNB recycling assay was performed after using 2-vinylpiyridine to remove the reduced GSH. Briefly, 2 µl of 2-vinylpiyridine and 4 µl of triethanolamine were mixed with 100 µl of TCA extract, followed by incubation in the dark at room temperature for 1 h before initiation of recycling assay. The increment in absorbance was converted to GSSG concentration using a GSSG standard curve. The GSH levels were calculated by subtracting the amount of GSSG formed from total GSH content obtained. The values were expressed as nmols GSH/mg protein.

### Detection of intracellular reactive oxigen species (ROS)

THP-1 cells resuspended in HBSS (Invitrogen, Carlsbad, CA) at 5×10^5^/mL were loaded with 2.5 µM of the membrane-permeable probe 5-(and-6)-chloromethyl-2′7′-dichlorodihydrofluorescein diacetate acetyl ester (CM-H_2_DCFDA, Molecular Probes, Eugene, OR) for 1 hour at 37°C. They were then washed with HBSS and stimulated with 6, 12, 25 µM DCE or CS or 500 µM diamide (positive control) and placed back into the incubator. After 30 minutes or 1 hour they were washed and resuspended in PBS. ROS generation was evaluated in flow cytometry (FACSCalibur; Becton Dickinson, San Jose, CA) by measuring the green fluorescence signal of DCF, the oxidation product of CM-H_2_DCFDA by free radicals. The emitted fluorescence was detected in Fl-1 using a 530/30 nm band-pass optical filter. FlowJo 8.8.2 software (Tree Star, Ashland, OR) was used to analyze data.

### HPLC analysis

10 µM DCE and CS were incubated with 5, 25 and 100 mM GSH in RPMI medium without FBS, at 37°C for 30 minutes and analysed by HPLC (Shimadzu, Kyoto, JP).

The reaction mixtures were charged on Mightysil RP-18 GP reverse-phase column, (150 mm×4.6 mm, Kanto Chemical, Tokyo, JP) maintained at 35°C and eluted with 35% acetonitrile at a flow rate of 1.0 ml/min. The elution profiles were monitored at 215 nm by UV detector. Retention times of DCE and CS peaks were 7.3 and 8.1 minutes, respectively.

### Immunoprecipitation and identification of glutathioylated proteins

Cells were lysed in RIPA buffer (20 mM TrisHCl, pH 8.0, 150 mM NaCl, 1% (wt/vol)Nonidet P-40, 1 mM EDTA, 10% glycerol, 100 mM NaF, 1 mM Na_3_VO_4_) supplemented with protease cocktail inhibitor for 15 minutes on ice with occasional vortexing. Equal amounts of proteins from the clarified cell lysates were incubated overnightat 4°C with rotation in the presence of 2 µg STAT3 antibody. The immune complexes were collectedby addition of protein A sepharose (Millipore Corp.), washed extensively, eluted in a non-reducing sample buffer (62.5 mM TrisHCl, pH 6.8, 10% glycerol, 5% SDS, 0.05% bromophenol blue) and separated on a 5% SDS–polyacrylamide gel. After electrophoresis, proteins were transferred to a PVDF membrane and nonspecific binding was blocked by incubation in 3% BSA diluted in TBST. Membranes were then probed with primary monoclonal antibodies against GSH (ViroGen, Watertown, MA) or STAT3. After washing, blots were incubated with anti-rabbit IgG peroxidase-conjugated antibody (Cell Signaling Technology). Protein-antibody reactions were detected with chemiluminescent detection system (Immun-Star™ WesternC™ Kit, Bio-Rad). The *S*-glutathionylated proteins on membranes were detected and quantified using the ChemiDoc XRS Imaging System (Bio Rad).

### Statistical analysis

Data are reported as means ± SD of four independent experiments (n = 4); statistical analyses were performed using Student's t test. Differences were considered significant whenp≤0.05.

## Results

### DCE and CS exert an inhibitory action on IL-6-elicited STAT3 in THP-1 cells

**Figure 2:**
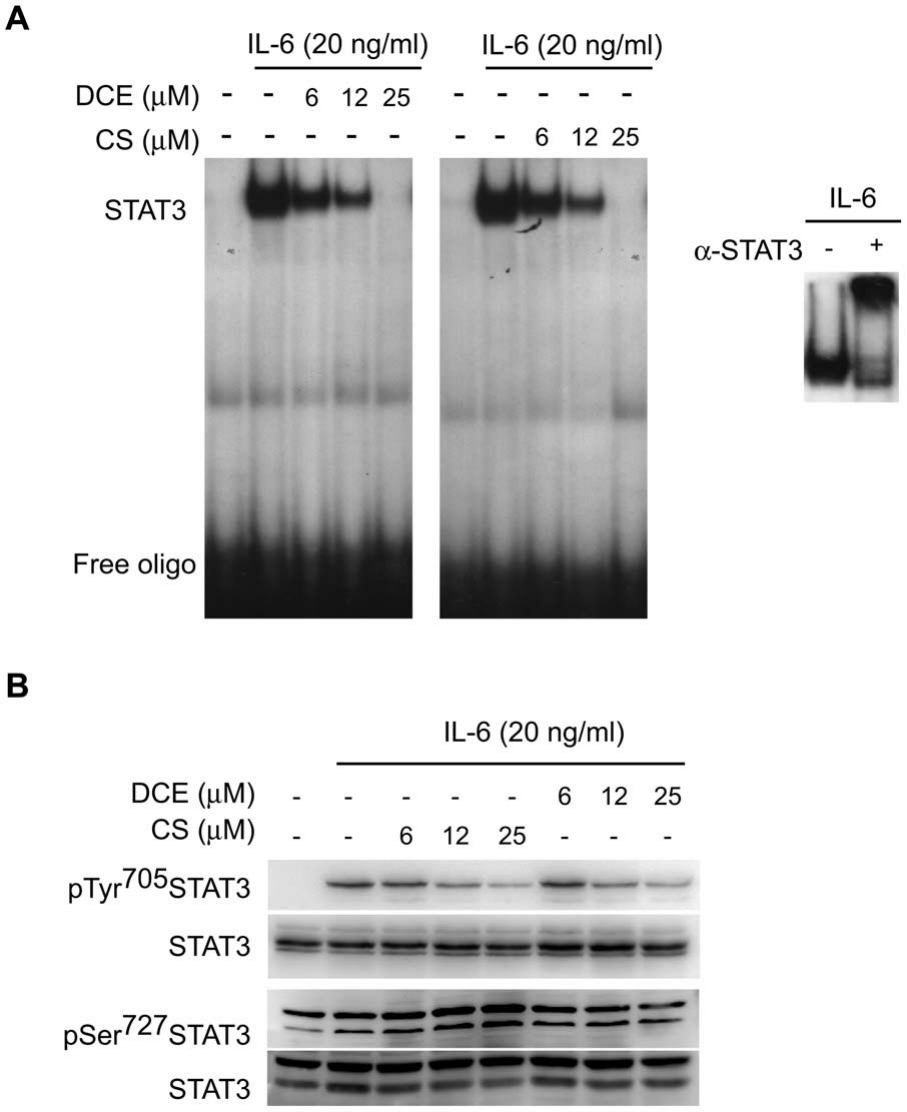
Effect of DCE and CS on IL-6-elicited STAT3 activation in THP-1 cells. (a) EMSA shows that DCE and CS dose-dependently decrease DNA-binding activity of STAT3 activated by 20 ng/ml IL6 in THP-1 cells. In the insert EMSA/supershift indicates that IL-6 induces prevalently the activation of STAT3. (b) Western Blot analysis shows that DCE and CS dose-dependently decrease tyrosine^705^ phosphorylation of STAT3 induced by 20 ng/ml IL-6 in THP-1 cells without changing the total amount of STAT3. Furthermore the sesquiterpens don't affect serine^727^ phosphorylation. The gels are representative of four independent experiments.

In order to evaluate the effect of DCE and CS on STAT3 signalling pathway, EMSA and Western blot analysis were performed in THP-1 cells treated with IL-6 (20 ng/ml) for 15 minutes. IL-6 increased predominantly the STAT3 DNA-binding activity, as indicated by EMSA/supershift experiment with anti-STAT3 antibody in line with previous report (Figure 2a). The administration of DCE and CS up to 25 µM 30 minutes before IL-6 treatment was able to inhibit in a concentration-dependent manner STAT3 DNA-binding activity with estimated EC_50_ value of 10 µM. (Figure 2a).

One of the critical steps leading to the activation of STAT3 is its phosphorylation on specific tyrosine residue and successive translocation into the nucleus. In line with above described data, Western Blot analysis showed that DCE and CS decreased, in a dose-dependent manner, IL-6-induced tyrosine^705^ phosphorylation of cytosolic STAT3 without affecting the total amount of STAT3 protein (Figure 2b). In addition to tyrosine phosphorylation, STAT3 is also phosphorylated on serine^727^ residue; as for others hematological cell line [35], also in THP-1 cells STAT3 is constitutively phosphorylated on serine residue and IL-6 only slightly induced it. DCE as well as CS were not able to modulate it (Figure 2b).

DCE and CS were also able to down modulate IL-6 dependent-tyrosine phosphorylation of STAT3 and its DNA binding activity in DLD-1 cell line (Figure S1).

### DCE and CS are able to inhibit IL-6-elicited JAKs phosphorylation

**Figure 3:**
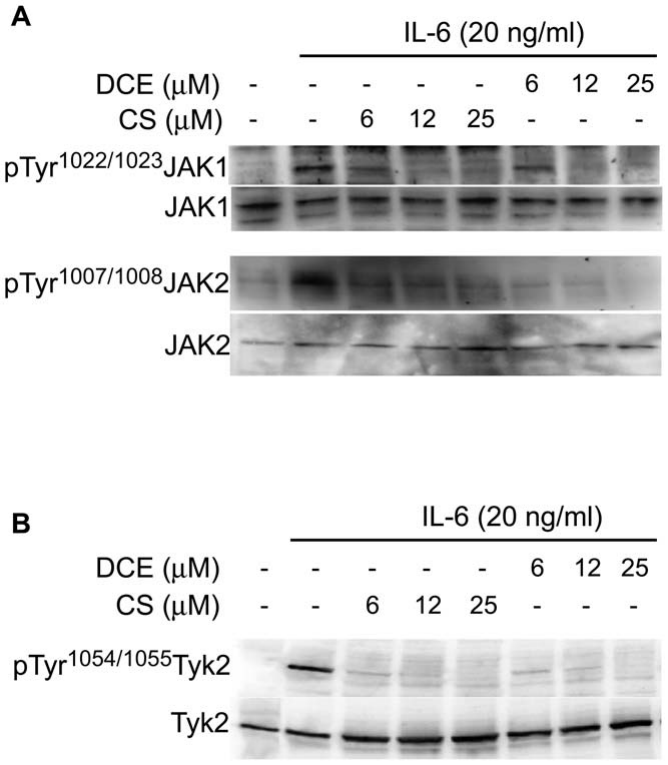
Effect of DCE and CS on tyrosine phosphorylation of JAK tyrosine kinases. Western Blot analysis shows that DCE and CS dose-dependentlydecreasetyrosine^1022/1023^ phosphorylation of JAK1 and tyrosine^1007/1008^ phosphorylation of JAK2 in THP-1 cells (a) and dose-dependently decreasetyrosine^1054/1055^ phosphorylation of Tyk2 in HeLa cells transiently transfected with Tyk2-pcDNA 3.0 (b). The compounds don't change the total amount of the corresponding non-phosphorylated proteins. The gelsarerepresentatives of four experiments performed separately.

The ability of DCE and CS to suppress the tyrosine phosphorylation of STAT3 suggests that these compounds may interfere with the function of upstream tyrosine kinases JAKs associated to the cytoplasmic portion of IL-6-receptor [36], [37]. Thus, we determined the effect of these terpenes on IL-6–induce tyrosine phosphorylation of JAK1 and JAK2 in THP-1 cells by Western Blot using antibodies that specifically recognizes phospho-Tyr^1022/1023^JAK1 and phospho-Tyr^1007/1008^JAK2. IL-6 induced the tyrosine-phosphorylation of both JAK1 and JAK2 in 10 minutes; notably, pre-treatment with 6–25 µM DCE or CS for 30 minutes resulted in almost complete dephosphorylation of both kinases. No change in total JAKs levels was observed (Figure 3a). Similar results were obtained in DLD-1 cell line (Figure S1). In order to analyse the effect of these terpenes on tyrosine^1054/1055^-phosphorylation of Tyk2, we used HeLa cell line transfected with Tyk2 expression plasmid. Western blot analysis showed that 6–25 µM DCE or CS induced almost complete dephosphorylation of IL-6-elicited Tyk2 in 30 minutes (Figure 3b).

Previous report described the inhibitory effect of DCE on constitutive STAT3 activation mediated by an increase in suppressor of cytokine signalling (SOCS) expression [31]. Treatment of THP-1 cells with 6–25 µM DCE or CS for 30 minutes to 1 hour had no impact on SOCS-3 expression (data not shown).

### Inhibition of the IL-6-elicited phosphorylation of STAT3 by DCE and CS is triggered by the drop in intracellular GSH level

In order to envisage the molecular mechanism of the inhibitory action of DCE and CS on STAT3 phosphorylation, we further hypothesized that the change in intracellular GSH level may play a critical role.

**Figure 4:**
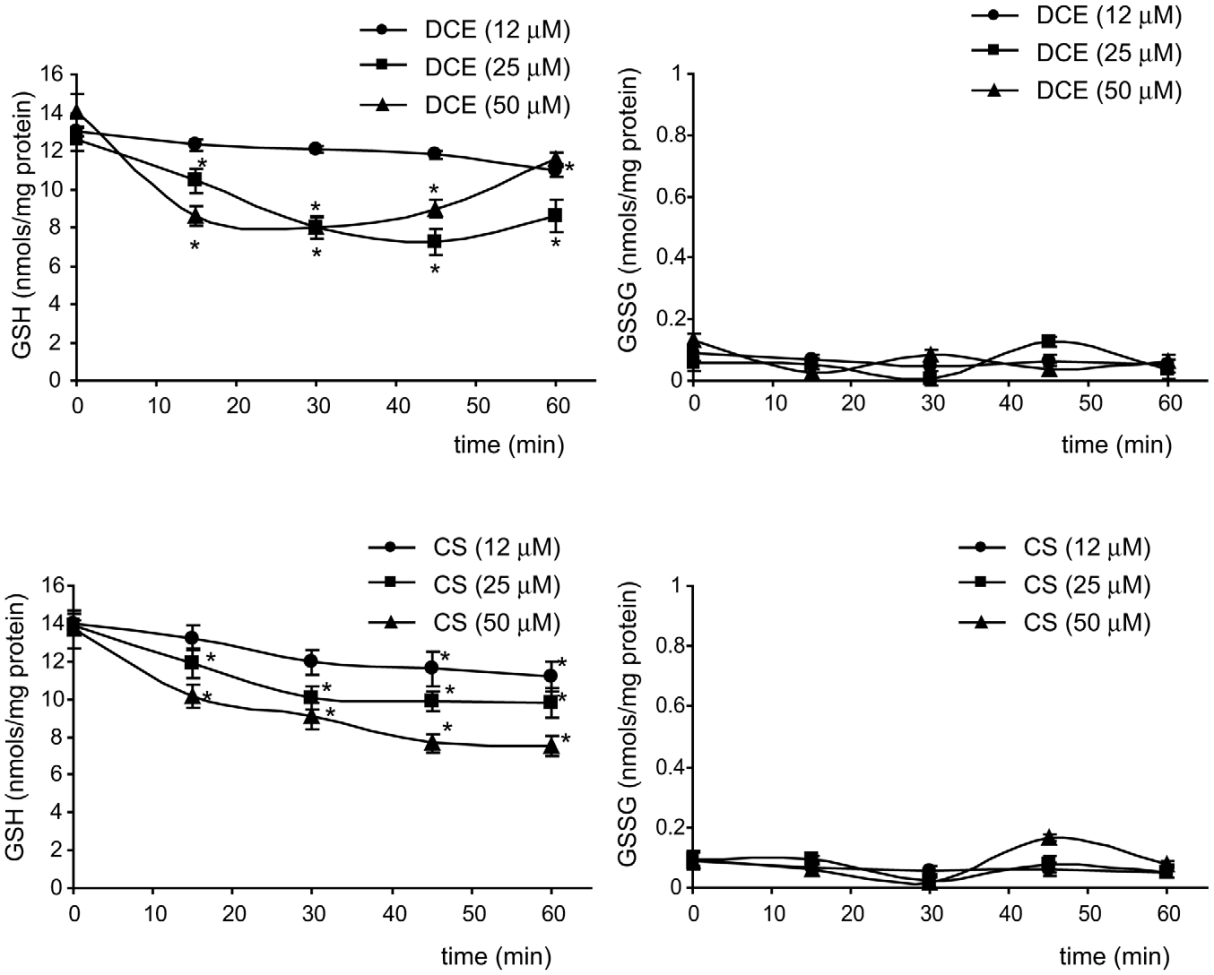
DCE and CS decrease intracellular GSH level in THP-1 cells. DCE and CS time- and dose-dependently induce the drop in cellular GSH content without significantly affecting the amounts of GSSG. Data are presented as means ± SD of results from four independent experiments. Significant difference compared to control group (*p≤0.05).

Spectrophotometric analysis measuring GSH and GSSG concentrations in THP-1 cells showed that under normal conditions the amounts of GSH (13±0.8 nmoles/mg protein) are far higher than those of GSSG (0.09±0.02 nmoles/mg protein) and that DCE and CS induced dose- and time-dependently the drop in GSH concentration, only slightly affecting GSSG level (Figure 4). The rapid drop in intracellular GSH amount is timely compatible with the inhibition of STAT3 phosphorylation.

**Figure 5:**
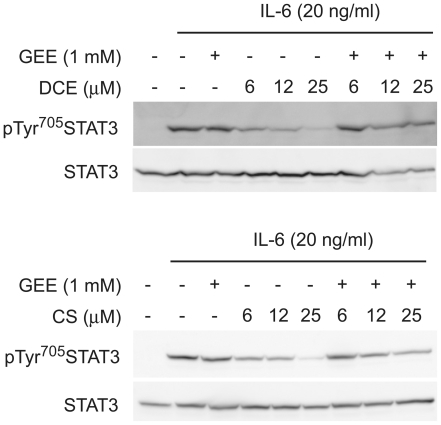
GEE reverts the inhibitory action of DCE and CS on the IL-6-elicited phosphorylation of STAT3. (a) Western Blot analysis shows that inhibitory action of DCE and CS on IL-6 induced STAT3 Tyr^705^ phosphorylation is reverted by 1 mM glutathione monoethyl ester (GEE). The total amount of STAT3 is not affected during the experiments. The gels are representative of four experiments performed separately.

To evaluate whether this event is crucial in the modulation of STAT3 pathway, THP-1 cells were pre-treated overnight with 1 mM glutathione monoethyl ester (GEE), the cell permeable GSH, and thereafter with the two sesquiterpene lactones up to 25 µM for 30 minutes before IL-6 administration. Western blot analysis showed that GEE reverts tyrosine-phosphorylation of STAT3 and this effect is correlated with the administrated sesquiterpenes' concentration (Figure 5). Also in DLD-1 cells, this post-translational modification is dependent on intracellular GSH concentration (Figure S1).

All these data further suggest the critical role played by intracellular GSH level in regulating IL-6-elicited-STAT3 activation.

### DCE and CS induce ROS production

**Figure 6:**
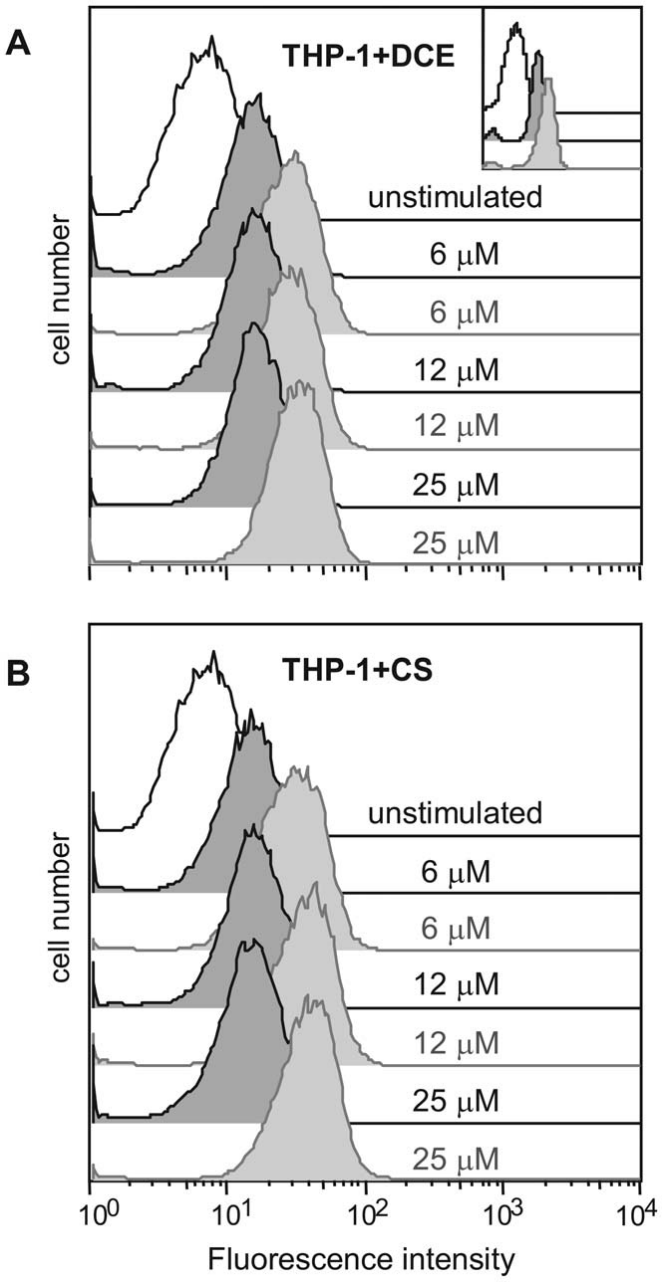
DCE and CS induce ROS production. Flow cytometric analysis of intracellular ROS generation. THP-1 cells loaded with CM-H_2_DCFDA and treated for 30 minutes (dark gray) and 1 hour (light gray) with 6, 12 and 25 µM (a) DCE or (b) CS shows and increment of fluorescence as respect to untreated cells (white). This increment is time-dependent but not dose-dependent. 500 µM diamide was used as positive control (inset). One representative experiment out of four is depicted.

THP-1 cells loaded with CM-H_2_DCFDA and treated with DCE or CS exhibited a clear fluorescence increase as respect to untreated sample. This data is consistent with an enhancement of intracellular ROS. Figure 6 shows that this effect is time-dependent but not dose-dependent. Indeed fluorescence intensity was similar at the different concentrations of the two compounds at the same time starting from 30 minutes to 1 hour stimulation. Likewise, diamide, a strong oxidant compound used as positive control, gave a time-dependent effect (inset).

### DCE and CS are able to directly interact with GSH

Previous works reported that some sesquiterpene lactones including CS, decrease the intracellular thiols level, without clear demonstration of the direct bond formation between them [24], [38].

**Figure 7:**
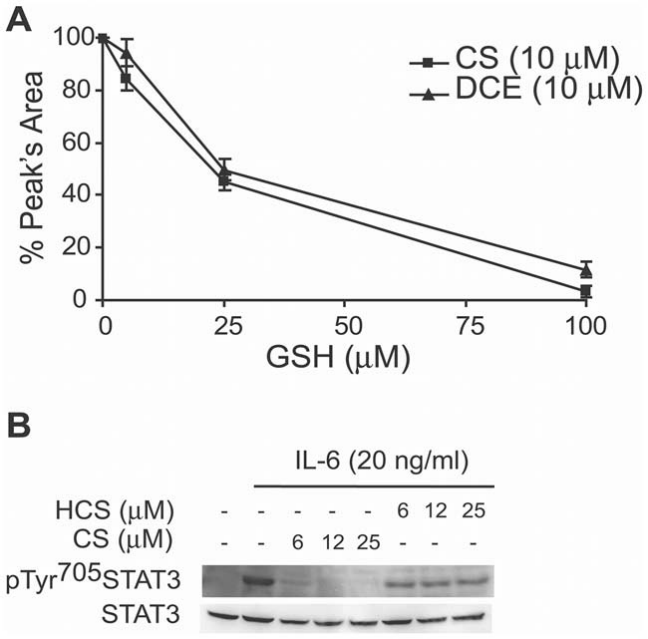
DCE and CS directly interact with GSH. (a) HPLC analysis shows that GSH dose-dependently decrease the amounts of DCE and CS after 30 minutes of incubation at 37°C. (Standard deviation of date obtained from three independent experiments is shown). (b) Western Blot analysis shows that HCS, is not able to decrease IL-6 induced STAT3 Tyr^705^ phosphorylation as CS does in THP-1 cells. The total amounts of STAT3 are not affected during the experiments. Data are the representatives of four experiments performed separately. The gels are representative of four experiments performed separately.

HPLC analysis of the solutions containing the sesquiterpene lactones plus GSH, prepared as described under Material and Methods, showed that the amounts of these terpenes decrease in the presence of increasing concentration of GSH, indicating their direct interaction(Figure 7a). These data suggest that the drop in intracellular GSH levels induced by DCE and CS may be mediated, at least partly, by this interaction.

DCE and CS, that contain an α-β-unsatured carbonyl moiety, function as potent Michael reaction acceptor. To analyze the importance of this group in the inhibitory action of these sesquiterpene lactones, we examined the effect of dehydrocostunolide (HCS), the reduced form of CS (Figure 1), on IL-6-elicited STAT3 phosphorylation. Contrary to DCE and CS, HCSfailed to exert any inhibitory action (Figure 7b).

### DCE and CS induce the S-glutathionylation of STAT3 interfering with its phosphorylation

A recent report describes that the strong oxidant diamide elicited *S*-glutathionylation of STAT3 leading to the inhibition of its tyrosine phosphorylation [20]. This post-translational modification may represent the mechanism of regulation of STAT3 activity in cells treated with DCE and CS.

**Figure 8:**
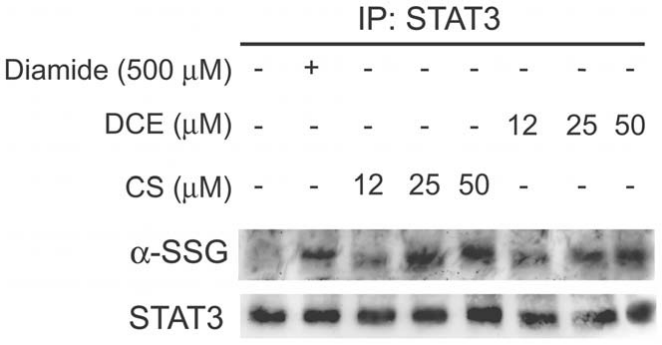
Effect of DCE and CS on glutathionylation of STAT3 in THP-1 cells. Western Blot analysis of immunoprecipitated STAT3 (IP STAT3) shows that DCE and CS dose-dependently increase the amounts of glutathionylated STAT3. At 50 µM both lactones induce the same level of STAT3 glutathionylation as that induced by 500 µM diamide, a strong oxidant. The total amounts of STAT3 are not affecting during the experiments. The gels are representative of four experiments performed separately.

As shown in Figure 8, the anti-glutathione antibody recognizes STAT3 immunoprecipitated proteins in THP-1 cells treated with 12–50 µM DCE and CS as well as with 500 µM diamide for 30 minutes, suggesting that these two sesquiterpene lactones induce dose-dependently the glutathionylation of STAT3. The blots exhibited equivalent STAT3 protein levels in all samples.

## Discussion

Receptor-associated JAK family tyrosine kinases, JAK1, JAK2 and Tyk2, plays a crucial role in the STATs signal transduction pathway activated by a number of cytokines and growth factors. Following the binding of these ligands to their specific membrane receptors, JAK kinases associated to cytoplasmic portion of receptors, tyrosine phosphorylate themselves and successively STAT proteins. Although this signalling pathway is finely regulated to guarantee the physiological response, hyper-activation of STATs has often been observed in a number of pathologies, especially that of STAT3 in cancer and in some inflammatory diseases. Therefore, attention has recently been paid to the compounds able to modulate STAT3 pathway in order to alleviate the detrimental effect of its hyper-activation.

The present study identifies two structurally related naturally occurring sesquiterpene lactones, DCE and CS, as inhibitors of IL-6-elicited STAT3 activationin human cell line THP-1 (EC_50_ = 10 µM) and demonstrates that DCE and CS rapidly suppress cytokine-induced tyrosine-phosphorylation of JAK1, JAK2 and Tyk2.

Previous report described the inhibitory effect of DCE on constitutive STAT3 activation mediated by an increase in SOCS expression [31]. The different proteins targeted by the same compound may reflect the diverse mode of activation of cytokines-induced and constitutively activated STAT3. In THP-1 cells, DCE and CS weren't able to modify the expression of SOCS-3 (data not shown). The rapid STAT3 inhibitory action showed herein seems to be more compatible with the notion that JAK family proteins, instead of SOCS-3, are their main target since the inhibition of the phosphorylation of pre-existing kinases may require far less time than de novo synthesis of SOCS-3 protein.

An increasing body of literature evidences underlines the critical involvement of intracellular redox state in cancer and in inflammation-associated diseases. Under normal conditions, mammalian cells contain 1–10 mM cytosolic GSH, depending on cell type and metabolic factors. GSH represents approximatively 95% of total non proteinthiols and is the main modulator of the cellular redox environment. The cytoplasmic high ratio between the reduced and oxidized glutathione (GSH/GSSG) is a key factor in keeping the cysteine residues of intracellular proteins in the reduced form. The decrease in GSH content, leading to the drop in the cellular redox potential, is often induced by oxidative stress. In the present study we show that, in line with above-described notion, in THP-1 cells GSH content is far higher than GSSG and that DCE and CS dose-dependently induce the consistent drop in intracellular GSH level without significantly affecting GSSG content (Figure 4). The drop in GSH content is timely compatible with the data on rapid inhibition of STAT3 phosphorylation by these compounds (Figure 4 and 2). The decrease in GSH concentration may be due to their ability both to generate oxygen species (ROS) [25], [26] and to interact with GSH. Data presented in Figure 6 show that DCE and CS induce time-dependent but not dose-dependent ROS production in THP-1 cells, suggesting that the drop in GSH content induced by these two compounds maybe due, at least in part, to their capacity to increase ROS production, in line with literature evidences [25], [26]. Results in Figure 7a furthermore indicate that DCE and CS are also able to directly interact with GSH. Since this interaction is shown to be highly efficient under our experimental conditions, it is supposed that two lactones elicit the rapid drop in the intracellular GSH content mainly through their capacity to interact with it.

The direct interaction between DCE or CS and GSH may be chemically mediated by the α-β-unsaturated carbonyl group present in their moiety (Figure 1), as described for others sesquiterpene lactones [39], [40]. This structural element may react with nucleophiles, such as cysteine sulfhydryl groups of cytosolic proteins, by a Michael-type addition, eventually leading to a mild electrophilic stress. Indeed, HCS, a structural analogue of CS lacking only the α-β-unsaturated carbonyl group (Figure 1), has failed to exert inhibitory action toward STAT3 tyrosine phosphorylation (Figure 7b), indicating that this terpenes' unsaturation may play a pivotal role in its biochemical activity. Furthermore, glutathione monoethyl ester (GEE), cell permeable form of GSH, reverts the inhibitory effect of these two sesquiterpene lactones on STAT3 phosphorylation (Figure 5), suggesting that their action is strictly correlated to the decrease in intracellular GSH concentration.

The disturbance in the GSH/GSSG homeostasis is implicated in the induction of reversibile*S*-glutathionylation of cysteine residues of sensitive proteins [18], [19]. Recently, STAT3 has been shown to be *S*-glutathionylated with concomitant loss of its phosphorylation in HepG2 cells treated with diamide, a strong oxidant compound, pointing out that this signal transcription factor is susceptible to redox regulation [20].

The present study showed that DCE and CS induce rapid *S*-glutathionylation of STAT3 with concomitant decrease in STAT3 tyrosine phosphorylation (Figure 8 and 2b). Since the *S*-glutathionylation of STAT3 induced by them is comparable to that induced by the strong oxidant, diamide [20] and the inhibition of STAT3 phosphorylation is reverted when cells are incubated with GEE (Figure 5), *S*-glutathionylation of STAT3 is likely to be triggered by increased oxidative stress induced by the decrease in GSH content. Although data presented in this study did not indicate the involvement of GSSG in this scenario due principally to the very low intracellular level of GSSG (Figure 4), its critical role in the glutathionylation of STAT3 cannot be excluded. To our knowledge, *S*-glutathionylation of STAT3 induced by naturally occurring phytocompounds has never been described so far.

We conclude that DCE and CS induce time- and dose-dependent drop in intracellular GSH content and consequently inhibit the tyrosine-phosphorylation of STAT3 in cells treated with IL-6. Enhanced oxidative pressure elicited either by direct interaction with GSH or by ROS generation may trigger an efficient STAT3 *S*-glutathionylation with concomitant decrease in tyrosine705 phosphorylation of STAT3. Dual action on STAT3 activation triggered, on the one hand, by the inhibition of JAKs catalytic activity that alone may lead to the decrease in STAT3 phosphorylation and, on the other hand, by *S*-glutathionylation of STAT3 that may lead to further interference on STAT3 phosphorylation seems to be compatible with an efficient action of DCE and CS.

Finally, considering the recent notion that STAT3 is a promising drug target in cancer prevention and/or therapy, the present work provides a base for the further structure/function study necessary for the future design/synthesis of DCE and CS-related compounds with stronger activity.

## Supporting Information

Figure S1 **Effect of DCE and CS on STAT3 activation in DLD-1 cells.** (a) EMSA shows that DCE and CS dose-dependently decrease DNA-binding activity of STAT3 activated by 20 ng/ml IL6 in DLD-1 cells. (b) Western Blot analysis shows that DCE and CS dose-dependently decrease tyrosine^705^ phosphorylation of STAT3 induced by 20 ng/ml IL-6 in DLD-1 cells without changing the total amount of STAT3. (c) DCE and CS slightly decrease tyrosine^1022/1023^ phosphorylation of JAK1 and tyrosine^1007/1008^ phosphorylation of JAK2 in DLD-1 cells. (d) Western Blot analysis shows that inhibitory action of DCE and CS on IL-6 induced STAT3 Tyr^705^ phosphorylation is reverted by 1 mM glutathione monoethyl ester (GEE). The total amount of STAT3 is not affected during the experiments.
(TIF)
