# Supplementary figures and images for: Two Naturally Occurring Terpenes, Dehydrocostuslactone and Costunolide, Decrease Intracellular GSH Content and Inhibit STAT3 Activation
Source: PLoS One. 2011 May 18;6(5):e20174. doi: 10.1371/journal.pone.0020174 (PMC3097233; doi:10.1371/journal.pone.0020174)

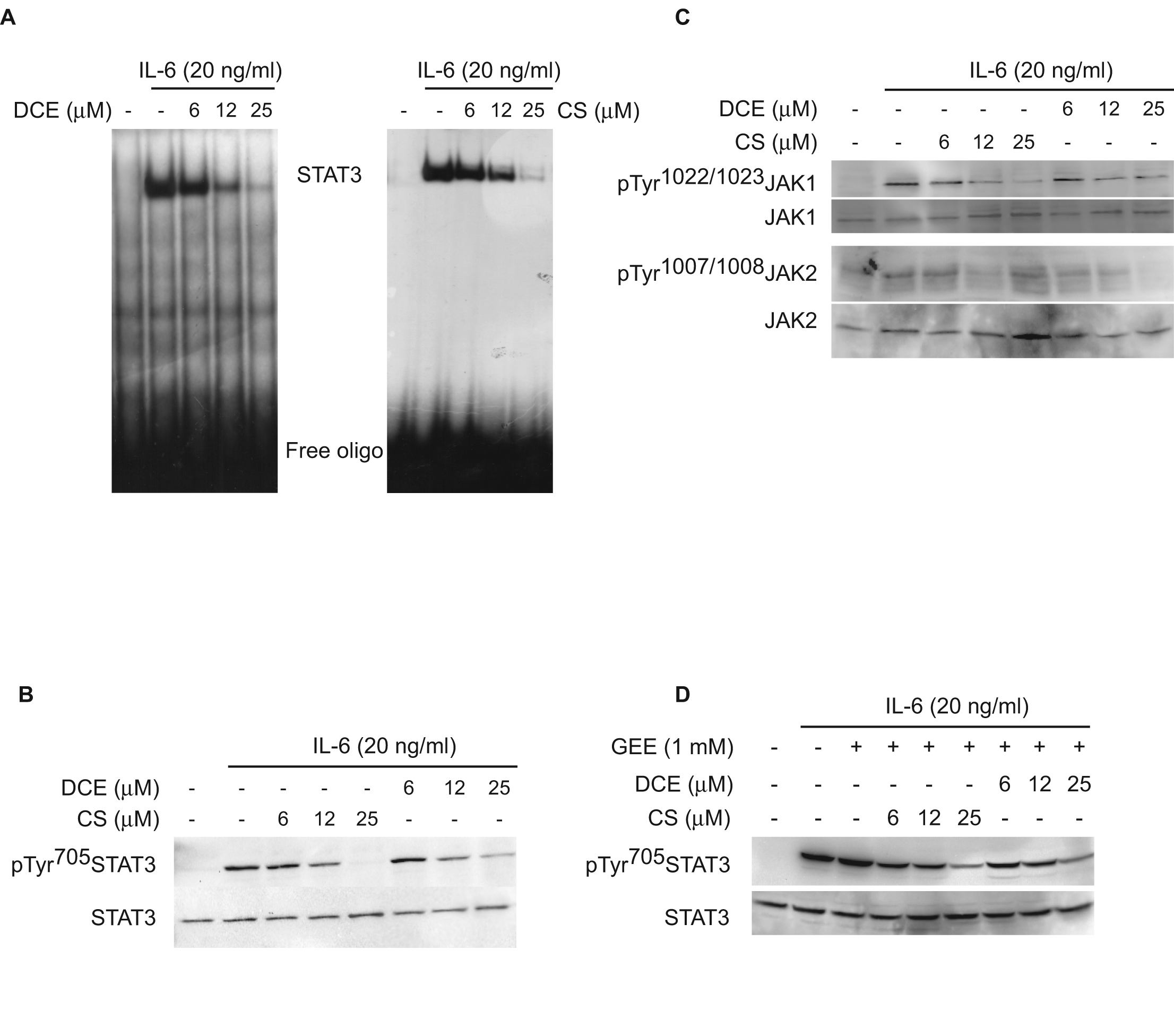

Supplement: Figure S1 — Effect of DCE and CS on STAT3 activation in DLD-1 cells. (a) EMSA shows that DCE and CS dose-dependently decrease DNA-binding activity of STAT3 activated by 20 ng/ml IL6 in DLD-1 cells. (b) Western Blot analysis shows that DCE and CS dose-dependently decrease tyrosine705 phosphorylation of STAT3 induced by 20 ng/ml IL-6 in DLD-1 cells without changing the total amount of STAT3. (c) DCE and CS slightly decrease tyrosine1022/1023 phosphorylation of JAK1 and tyrosine1007/1008 phosphorylation of JAK2 in DLD-1 cells. (d) Western Blot analysis shows that inhibitory action of DCE and CS on IL-6 induced STAT3 Tyr705 phosphorylation is reverted by 1 mM glutathione monoethyl ester (GEE). The total amount of STAT3 is not affected during the experiments. (TIF) [file pone.0020174.s001.tif]
